# Myeloid-Derived Suppressor Cells Participate in Preventing Graft Rejection

**DOI:** 10.1155/2012/731486

**Published:** 2012-03-15

**Authors:** Yan Wang, Xiaodong Gu, Jianbin Xiang, Zongyou Chen

**Affiliations:** Department of General Surgery, Huashan Hospital, Fudan University, Shanghai 200040, China

## Abstract

Myeloid-derived suppressor cells (MDSCs) are a heterogeneous population of cells and have a tremendous potential to suppress immune responses. MDSCs accumulate during tumor progression, autoimmunity, chronic infection, transplantation, and other pathological conditions and can potently suppress T-cell function. Here, we discuss recent findings that describe the molecular mechanisms of MDSCs suppressing T-cell immune responses as well as recent observations that MDSCs may have roles in transplant tolerance.

## 1. Introduction

Immature myeloid cells (IMCs) are part of the normal process of myelopoiesis, which takes place in the bone marrow and is controlled by a complex network of soluble factors. Haematopoietic stem cells differentiate into common myeloid progenitor cells and then into IMCs [1]. In normal individuals, IMCs migrate into different peripheral organs, where they quickly differentiate into macrophages, dendritic cells, or granulocytes. However, factors that are produced during acute or chronic infections, trauma, or sepsis and in the tumor microenvironment promote the accumulation of IMCs at these sites, prevent their differentiation, and induce their activation. These cells exhibit immunosuppressive functions and are therefore known as myeloid-derived suppressor cells (MDSCs) [2]. MDSCs are not a defined subset of myeloid cells but rather a heterogeneous population of activated IMCs that have been prevented from fully differentiating into mature cells. MDSCs lack the expression of cell-surface markers that are specifically expressed by monocytes, macrophages, or dendritic cells and comprise a mixture of myeloid cells that have the morphology of granulocytes or monocytes. Early studies showed that 1–5% of MDSCs can form myeloid cell colonies and that about one-third of this population can differentiate into mature macrophages and dendritic cells in the presence of the appropriate cytokines in vitro and in vivo [3].

MDSCs are a heterogeneous population of cells that consist of myeloid progenitors and immature macrophages, immature granulocytes, and immature dendritic cells [4]. MDSCs were first characterized more than 20 years ago in tumor-bearing mice and in patients with cancer [5]. There are many tumor-derived factors that can promote the expansion of MDSCs through the stimulation of myelopoiesis and inhibit the differentiation of mature myeloid cells, such as vascular endothelial growth factor (VEGF), prostaglandin E2 (PGE2), granulocyte-macrophage colony-stimulating factor (GM-CSF), transforming growth factor-*β* (TGF-*β*), interleukin- (IL-) 1*β*, IL-10, IL-6, and macrophage colony-stimulating factor (M-CSF) [6]. Glucocorticoids are also believed to have inhibitory effects on the maturation of IMCs. Most tumor-derived factors exert the inhibiting effects on differentiation and maturation of myeloid cells through signal transducer and activator of transcription 3 (STAT3) signaling pathway [7]. In animal tumor models and cancer patients, MDSCs, induced by tumor-derived factors, accumulate in large numbers in the blood, bone marrow, spleen, and tumor masses, mediating the downregulation of T-cell immunity, thus leading to tumor escape, progression, and metastasis [8].

Although initial observations and most of the current information on the role of MDSCs in immune responses have come from studies in the field of cancer research, accumulating evidence has shown that MDSCs also regulate immune responses during infectious diseases, autoimmune disorders, and transplantation [9–14]. Evidence for a role of MDSCs in transplantation is emerging from various animal models. An expansion of MDSCs was first described in a rat model of kidney allograft tolerance induced by anti-CD28 antibodies [15]. Recently, MDSCs have been considered a key role in several transplantation models, and study on the mechanism of MDSC-induced immune suppression may generate new insights into our understanding of allograft tolerance and improve therapeutic efficiency in transplantation. In this paper, we discuss the phenotype and subsets, the mechanisms of suppressive function of MDSCs, and the possible role of these cells in organ transplantation.

## 2. Phenotype and Subsets of MDSCs

MDSCs represent a heterogeneous population of myeloid cells at different stages of differentiation that comprises myeloid progenitor cells and immature myeloid cells (macrophages, granulocytes, and dendritic cells). There is no strict cell-surface-marker-guided classification of MDSC available at present (Table 1).

In mice, MDSCs are commonly identified as the cell membrane that simultaneously expresses two markers: one is CD11b, an adhesion molecule also known as Mac-1, the other is Gr1 antigen, a 21–25 kDa glycosylphosphatidylinositol- (GPI-) anchored protein. Normal mouse bone marrow contains 20–30% of cells with this phenotype, but these cells make up only a small proportion (2–4%) of spleen cells and are absent from the lymph nodes [32]. More recently, according to MDSCs morphological and functional features, as well as their expression of the two molecules lymphocyte antigen 6 complex, locus C(Ly6C) and lymphocyte antigen 6 complex, locus G(Ly6G), MDSCs were subdivided into two different subsets of granulocytic MDSCs (CD11b+ Ly6G+ Ly6Clow) and monocytic MDSCs (CD11b+ Ly6G− Ly6Chigh) [33]. In addition to CD11b and Gr1, MDSCs express additional markers of early myeloid differentiation, such as CD31, ER-MP54, and ER-MP58, and low levels of costimulatory molecules [34]. Some researchers also identified a more specific population of MDSCs that express Gr1 and CD115, which has much stronger suppressive activity compared with the classic Gr1+ CD11b+ MDSCs [35].

In humans, MDSCs are even less well defined owing to the lack of specific markers. Human cells do not express a marker homologous to mouse Gr1. MDSCs are most commonly defined as CD14-CD11b+ cells or, more narrowly, as cells that express the common myeloid marker CD33 but lack expression of markers of mature myeloid and lymphoid cells and of the MHC class II molecule HLA-DR [36]. MDSCs have also been identified within a CD15+ population in human peripheral blood. In healthy individuals, IMCs constitute *∼*0.5% of peripheral blood mononuclear cells. MDSCs in human were also subdivided into two subsets: granulocytic MDSCs express CD15+ CD33+ CD11b+ with minimal or no HLA-DR expression, while monocytic MDSCs express CD14 with minimal or no HLA-DR expression, CD49d (also known as integrin *α*4) and low levels of CD15 [37].

The terminally differentiated granulocytic MDSCs represent 70–80% of MDSCs. Monocytic MDSCs, accounting for 20–30% of MDSCs, retain the ability to differentiate into mature dendritic cells and macrophages (Table 2). Although these subsets can have various functions and distributions depending on their environment, their capacity to induce T-cell hyporesponsiveness is generally considered equal [38].

## 3. Suppressive Function of MDSCs

A growing body of evidence suggests that MDSCs have a remarkable suppressive effect on T-cell proliferation. Most studies have shown that the immunosuppressive functions of MDSCs require direct cell-cell contact, which suggests that they act either through cell-surface receptors or through the release of short-lived soluble mediators [4]. Here, we will elaborate the mechanismsby whichMDSCs suppress T-cell responses and the effects of MDSCs in organ transplantation.

### 3.1. Arginase-1 (Arg-1) and Inducible Nitric Oxide Synthase (iNOS)

Historically, the suppressive activity of MDSCs has been associated with the metabolism of L-arginine. L-arginine serves as a substrate for two enzymes, iNOS (which generates NO) and Arg-1 (which converts L-arginine to urea and L-ornithine). MDSCs express high levels of both Arg-1 and iNOS, and a direct role for both of these enzymes in the inhibition of T-cell function is well established [39].

Although the first experiments underlying the importance of L-arginine metabolism in cancer were performed more than 50 years ago, only recently has the role of Arg-1 in tumor growth and escape from the immune surveillance been clarified [40]. Arg-1 can be released or expressed by either cancer cells or tumor-associated myeloid cells, including putative MDSCs. Recent data suggest that there is a close correlation between the availability of L-arginine and the regulation of T-cell proliferation. The increased activity of Arg-1 in MDSCs leads to enhanced L-arginine catabolism, which depletes this nonessential amino acid from the microenvironment. The shortage of L-arginine inhibits T-cell proliferation through several different mechanisms, including decreasing their expression of CD3 *ζ*-chain and preventing their upregulation of the expression of the cell cycle regulators cyclin D3 and cyclin-dependent kinase 4 [41]. An expansion of MDSCs was detected in immunoglobulin-like transcript 2 (ILT2) transgenic mice [28]. In this model, adoptive transfer of MDSCs from ILT2 mice significantly delayed the rejection of major MHC-II-mismatched skin allografts. This effect was associated with a unique MDSCs transcriptional profile including upregulation of Arg-1, but not iNOS. Highfill et al. [42] found that exogenous IL-13 produced an MDSCs subset that was more potently suppressive and resulted in Arg-1 upregulation. These MDSCs were more effective to inhibit graft-versus-host disease (GVHD). GVHD inhibition was reduced when Arg-1 deficient MDSCs were used.

iNOS can be induced in myeloid cells by different tumor-secreted factors such as VEGF, GM-CSF, and IL-6. MDSCs expressing iNOS can inhibit mitogenic and peptide-specific responses through NO production. MDSC-mediated T-cell inhibition is associated with the impairment of the main signaling pathways coupled to the IL-2 receptor as demonstrated by the lack of JAK3, STAT5, extracellular signal-regulated kinase, and Akt phosphorylation in response to IL-2 [43]. NO is able to induce a reversible type of T-cell anergy by reducing phosphorylation of tyrosine residues on JAK3 and STAT5. NO also can reduce MHC-II expression, either by downregulating IFN-*γ*-induced expression of class II transactivator or by inhibiting DNA binding of transcription factor NF-Y at the class II promoter Y box [44]. MHC-II expression is critical for antigen-specific immunity. In a model of MHC-mismatched rat kidney allograft, treatment with anti-CD28 antibodies induced long-term survival and was associated with the presence, in tolerated allografts, of MDSCs that operated through iNOS activity [15]. The action of NO production was critical to the immunosuppression mediated by MDSCs and in maintaining the tolerant state in vivo. In this kidney transplantation model, the injection in tolerant animals of amino guanidine, which inhibits iNOS, broke the established tolerance and led to graft rejection. These results suggest that MDSCs, accumulated in the blood of tolerant kidney recipients, release high levels of NO after contact with activated effector T cells and specifically control their proliferative response.

### 3.2. Heme Oxygenase-1 (HO-1)

HO-1 catabolizes pro-oxidant heme groups into carbon monoxide, biliverdin and ferritin, three metabolites involved in immunoregulatory processes [45, 46]. Recently, De Wilde et al. [29] reported the observation of HO-1-dependent MDSCs-mediated alloreactive T-cell suppression, which was cell-to-cell contact dependent and requires IL-10 activity. They found that transfer of MDSCs from LPS-treated mice in untreated recipients significantly prolonged skin allograft survival. To specifically address the role of HO-1 in this MDSCs-mediated delay of allograft rejection was tested by incubating purified MDSCs with the HO-1-specific inhibitor SnPP pretreatment before an adoptive transfer in female mice. SnPP treatment of MDSCs abrogated the inhibition of allograft rejection. This demonstrates that HO-1 activity is a dominant effector of in vivo immune suppression mediated by MDSCs.

### 3.3. Radical Oxygen Species (ROS)

The production of ROS also contributes to the suppressive activity of MDSCs, as increased ROS levels in MDSCs induce the upregulation of several subunits of the NADPH oxidase [47]. ROS can induce DNA damage in immune cells resident in the tumor microenvironment, inhibit the differentiation of MDSCs into functional dendritic cells, and recruit MDSCs to the tumor site. Moreover, extracellular ROS catalyzes the nitration of the TCR, which consequently inhibits the T-cell-peptide-MHC interaction resulting in T-cell suppression [48]. The involvement of ROS in the suppressive activity of MDSCs is not restricted to neoplastic conditions. Indeed, inflammation and microbial products are also known to induce the development of an MDSC population that produces ROS following its interaction with activated T cells.

### 3.4. Regulatory T Cells (Treg)

Recently, MDSCs have been shown to enhance the development of Treg, possibly through interactions between CD80 expressed by MDSCs and CTLA-4 expressed by Treg, production of IL-10, and/or preferential inhibition of activated T cells through NO [35, 49]. In a mouse model of lymphoma, MDSCs were shown to induce Treg expansion through a mechanism that involved Arg-1 and the capture, processing, and presentation of tumor-associated antigens by MDSCs but was independent of TGF-*β* [50]. In a mice model of skin transplantation, recipents were injected with recombinant G-CSF, or IL-2 complex(IL-2C), Gr1+ CD11b+ MDSC or CD4+ Foxp3+ Treg were induced in circulation of recipients [27]. They found that although treatment with either IL-2C or G-CSF led to a significant delay of MHC-II disparate allogeneic donor skin rejection, the combinatorial treatment was superior to either alone, confirming that MDSCs and Treg prolonged skin allograft survival in mice. Karp and Mannon [51] summarized identified an HLA-Dq*α*-class-II-derived peptide that was a potent inducer of CD11b+CD115+Gr1+ MDSCs. Moreover, this peptide prolonged the survival of fully mismatched mouse cardiac allografts associated with the induction of Foxp3+ Treg. Depletion or inhibition of function of MDSCs reversed the prolonged survival and decreased Treg in the recipient.

### 3.5. CD8+ T Cells

MDSCs can take up soluble antigens, including tumor-associated antigens, and process and present them to T cells. Blocked MDSCs-T cells interactions with a MHC-I specific antibody abrogate MDSC-mediated inhibition of T-cell responses in vitro. The MHC-I restricted nature of MDSC-mediated CD8+ T cell suppression has also been demonstrated in vivo in tumor models [52, 53]. MDSCs can abrogate the expression of L-selectin on CD8+ T-cell, suppressing the homing of these cells to the tumor site, where they would be activated. MDSCs cleave L-selectin from T cells because they constitutively express ADAM17 at their cell surface and, as a result, T cells cannot traffic to tumor draining lymph nodes, where they normally would have access to tumor antigens and consequently can not be activated [54]. One study showed a potential mechanism for IL-10- and IFN-*γ*-dependent MDSCs regulation of CD8+ T cell function mediated through programmed cell death-1 (PD-1) and PD-1 ligand interaction [55]. They testified the PD-1 signaling pathway inducing the apoptosis of CD8+ T cells and phagocytosed CD8+ T cells, contributing to CD8+ T cell exhaustion. However, whether PD-1 signaling pathway plays a role in MDSCs-mediated T cell suppression remains controversial, and further investigations are needed.

### 3.6. T-Helper 2 (Th2) Cells

A recent study found that MDSCs can impair tumor immunity not only by suppressing T-cell activation but also by interacting with macrophages to increase IL-10 and decrease IL-12 production, thereby promoting a tumor-promoting type 2 response [56]. Many literatures have reported that MDSCs inhibit antigen-specific and nonspecific T-cell functions via several different mechanisms, including Arg-1, NO, ROS, IL-10, and TGF-*β* [35]. Furthermore, using a depleting antibody, Delano et al. [57] demonstrated that expansion of MDSCs in vivo contributed to the induced Th2 polarization of antibody responses after sepsis. Challenging mice with T-cell-dependent antigens, such as NP-KLH, offers the opportunity to explore in vivo the shift in antibody class switching to IgG_2a_ or IgG_1_ production, which is dependent on cytokines, including IFN-*γ* and IL-4, and reflects this predilection toward a Th2 versus a Th1 CD4+ T-cell response. Turnquist et al. [31] found that IL-33 administration greatly increased splenic MDSCs in normal and transplanted mice. It has been suggested that IL-33 prolongs cardiac allograft survival by promoting Th2 responses. Administration of IL-33 concurrent with cardiac allotransplantation increased systemic levels of IL-5 and IL-13, increased IL-5+CD4+ cells, and decreased CD8+INF-*γ*+ T cells. Notably, IL-13 is implicated in tolerance, particularly by targeting myeloid cells and activating the suppressive function of MDSCs.

## 4. Summary

MDSCs aid tumor development by exerting a profound inhibitory activity on T cells. The mechanism of MDSCs possessing a direct role in the inhibition of T-cell function is well established in tumors. Their potential role in organ transplantation requires far more investigation. Recently, Qian's group have found that cotransplantation with in vitro generated MDSCs can effectively protect islet allografts from host immune attack [30]. Our study also demonstrated that MDSCs can be propagated in vitro from bone-marrow-derived myeloid precursor cells under the influence of hepatic stellate cells. Adoptive transfer of these in vitro generated cells can prolong cardiac allograft survival. However, the mechanism of MDSCs causing immunosuppression in this model has not yet been explored. A detailed understanding of MDSCs regulation of T-cell immune function in transplantation will undoubtedly lead to the design of more effective strategies to achieve transplant tolerance in the clinic.

## Figures and Tables

**Table 1:** Summary of phenotype of MDSCs in tumors and transplantation models.

Tumour type	Reported phenotype	References
Renal cell carcinoma	CD11b^+^/CD14^−^/CD33^+^/CD15^+^	
	CD66b^+^/VEGF1^+^	Rodriguez et al. [16]
Non small cell lung cancer	CD11b^+^/CD14^−^/CD15^+^/CD33^+^	Liu et al. [17]
Colon carcinoma	Lin^−^/HLA-DR^−^/CD33^+^/CD11b^+^	Diaz-Montero et al. [18]
Breast carcinoma	Lin^−^/HLA-DR^−^/CD33^+^/CD11b^+^	Diaz-Montero et al. [18]
Prostate cancer	CD14^+^/HLA-DR^low/neg^	Vuk-Pavlović et al. [19]
Malignant melanoma	CD14^+^/CD11b^+^/HLA-DR^low/neg^	Filipazzi et al. [20]
	CD80^+^/CD86^+^	Poschke et al. [21]
Hepato-cellular carcinoma	CD14^+^/HLA-DR^low/neg^	Hoechst et al. [22, 23]
Hodgkin lymphoma (HL)	CD11b^+^, CD13^+^, CD34^+^, CD14-, CD45+	Parrinello et al. [24]
Non hodgkin lymphoma	CD14^+^/HLA DR^low/neg^/CD120^low^	Lin et al. [25]
Myelodysplastic syndrome (MDS)	LIN^−^/HLA-DR^−^/CD33^+^	Wei et al. [26]

Transplantation model	Reported phenotype	References

Rat kidney allograft	CD11b^+^CD6^−^CD80/86^+^NKRP-1^+^	Dugast et al. [15]
Mouse skin allograft	CD40^+^/CD80^+^/F480^+^/IL-4R*α* ^+^	Adeegbe et al. [27]
	CD11b^+^/Gr1^+^	Zhang et al. [28],
		De Wilde et al. [29]
Mouse hepatic islet allograft	CD11b^+^/CD45^+^	Chou et al. [30]
Mouse cardiac allograft	CD11b^+^/Gr-1^int^/F4/80^+^	Turnquist et al. [31]

**Table 2:** Phenotype of monocytic and granulocytic MDSCs subsets in murine and human.

	Granulocytic MDSCs	Monocytic MDSCs
Murine	CD11b^+^ Ly6G^+^ Ly6C^low^Gr-1^high^CD49d^−^	CD11b^+^ Ly6G^−^Ly6C^ high^Gr-1^int⁡^CD49d^+^
Human	MHC class II^low^CD33^+^CD11b^+^ CD14^−^CD15^+^	MHC class II^low^CD33^+^CD11b^+^ CD14^+^CD66b^+^
